# Effectiveness of Propofol versus Dexamethasone for Prevention of Postoperative Nausea and Vomiting in Ear, Nose, and Throat Surgery in Tikur Anbessa Specialized Hospital and Yekatit 12th Hospital, Addis Ababa, Ethiopia

**DOI:** 10.1155/2020/4258137

**Published:** 2020-09-07

**Authors:** Abere Tilahun Bantie, Wosenyeleh Admasu, Sintayehu Mulugeta, Abera Regassa Bacha, Desalegn Getnet Demsie

**Affiliations:** ^1^Department of Anesthesiology, College of Medicine and Health Sciences, Adigrat University, Adigrat, Ethiopia; ^2^School of Anesthesiology, College of Health Sciences, Addis Ababa University, Addis Ababa, Ethiopia; ^3^Department of Anesthesiology, College of Health Sciences, Mekelle University, Mek'ele, Ethiopia; ^4^Department of Anesthesiology, College of Medicine and Health Sciences, Axum University, Axum, Ethiopia; ^5^Department of Pharmacy, College of Medicine and Health Sciences, Adigrat University, Adigrat, Ethiopia

## Abstract

**Background:**

Postoperative nausea and vomiting (PONV) remain as common and unpleasant and highly distressful experience following ear, nose, and throat surgery. During ENT surgery, the incidence of PONV could be significantly reduced in patients who receive dexamethasone and propofol as prophylaxis. However, the comparative effectiveness of the two drugs has not been assessed. The aim of this study was to compare the effectiveness of propofol and dexamethasone for prevention of PONV in ear, nose, and throat surgery.

**Methods:**

This study was conducted in 80 patients, with ASA I and II, aged 18–65 years, and scheduled for ENT surgery between December 20, 2017, and March 20, 2018. Patients were randomly assigned to Group A and Group B. Immediately after the procedure, Group A patients received single dose of intravenous (IV) dexamethasone (10 mg/kg) and Group B patients were given propofol (0.5 mg/kg, IV), and equal follow-up was employed. The incidence of PONV was noted at 6th, 12th, and 24th hour of drug administration. Independent *t*-test and Mann–Whitney test were used for comparison of symmetric numerical and asymmetric data between groups, respectively. Categorical data were analyzed with the chi-square test, and *p* value of < 0.05 was considered as level of significance.

**Results:**

The incidences of PONV throughout the 24-hour postoperative period were 35% in the propofol group and 25% in the dexamethasone group. Statistical significance was found in incidence of PONV (0% versus 22.5%) and use of antiemetic (0% versus 5%) between dexamethasone and propofol groups, respectively, at 12–24 hours. Over 24 hours, 5% in dexamethasone group and 12.5% in propofol group developed moderate PONV, while none of the participants felt severe PONV.

**Conclusions:**

Dexamethasone was more effective than propofol to prevent PONV with lower requirements of rescue antiemetics.

## 1. Introduction

Postoperative nausea and vomiting (PNOV) remain common and distressing complication following ear, nose, and throat (ENT) surgery, especially when no prophylaxis is given [1–6].

The pathophysiology of PONV is multifactorial; multiple pathways, neurotransmitters, and risk factors are involved. Some of the contributing factors for PONV include age < 50 years, female gender, history of previous PONV or motion sickness, nonsmoking, obesity, surgical- and anaesthetic-related factors, and/or parental anxiety [7–9].

The feelings associated with PONV are unpleasant and distressful requiring multimodal treatment approaches [7, 8]. Recent evidence indicated that inadequate prevention or treatment of PONV potentiates prolonged recovery and hospitalization, unpleasant hospital experiences, and increased health care costs [10, 11]. For instance, prolonged vomiting may result in electrolyte imbalance (hypocalcaemia, hypochloremia, and hyponatremic metabolic alkalosis) and dehydration, Mallory-Weis tears, esophageal rupture, aspiration, postoperative bleeding, and airway obstruction especially in patients undergoing ENT surgery [12].

The use of antiemetics can reduce the occurrence of PONV from over 52% to less than 30% in certain populations [1, 13]. To decrease the incidence of PONV, a number of antiemetics including antihistamines [14], butyrophenones [14, 15], serotonin receptor antagonists, corticosteroids [16], and anaesthetic agents [17] have been tried in clinical use. Nevertheless, most of the antiemetics are associated with undesirable adverse effects, such as sedation, hypotension, dysphoria, dry mouth, restlessness, and extrapyramidal symptoms.

Several studies have shown that dexamethasone, a corticosteroid, is an effective antiemetic for PONV prophylaxis in various types of surgery and in improving surgical outcomes [18, 19]. Propofol, an antagonist at the 5-HT3 receptor, is also a novel total intravenous anaesthetic that possesses antiemetic properties when given in subhypnotic doses as part of combination therapy [5, 20]. Low-dose intravenous propofol (0.5 mg/kg) is effective for prevention of PONV with no significant complications [4, 21, 22]. Even though propofol has been used by a number of anesthesiologists, it is still under investigation. Thus, the aim of this study was to compare the effectiveness of dexamethasone and propofol for the prevention of PONV in ENT surgery.

## 2. Methods

Ethical approval was obtained from Addis Ababa University, Ethics Committee, and assigned an ethical approval, No-98/2010 on December 11, 2018. All patients who were scheduled to undergo elective ENT surgery at Tikur Anbessa Specialized Hospital (TASH) and Yekatit Hospital from December 20, 2017, to March 30, 2018, were enrolled in the study.

All patients of both sexes who had elective ENT surgery under general anesthesia, with ASA I and II, and aged between 18 and 65 years were included in the study. Patients requiring admission to the intensive care unit or mechanical ventilation, premedicated with emetogenic or antiemetic agents, with previous history of nausea/vomiting, with hypotension, gastroesophageal reflux disorder, and insulin-dependent diabetes, and smoking were excluded from the study. Two-independent-sample size formula was used to determine the sample size for each group based on the mean difference of the visual analog score. A total of 80 patients with an American Society of Anesthesiologists physical status I and II and age of 18–65 years were assigned to each group. Afterwards, patients were assigned to either Group A (dexamethasone, *n* = 40) or Group B (propofol, *n* = 40) randomly by lottery method from daily schedule list.

Following preoperative preparation, all elective ENT surgery scheduled patients who fulfilled inclusion criteria and volunteered to take part in the study were oriented by trained data collectors on how to self-report nausea using the eleven-point numeric rating score (NRS) from score 0 to 10. Bachelor's and master's anesthetists were responsible for carrying out all anesthesia management. After checking baseline vital signs and achieving adequate preoxygenation for five minutes, patients in both Group A and Group B were induced with intravenous 3–5 mg/kg thiopentone, 2 *µ*g/kg fentanyl, and 2 mg/kg suxamethonium. Then anesthesia was maintained with 0.75–1.5% of halothane with 4 L/min flow of 100% oxygen and intermittent vecuronium (0.04 mg/kg). At the end of the procedure, patients were fully reversed with 0.02 mg/kg atropine and 0.04 mg/kg neostigmine. Immediately after extubation, Group A patients received a single dose of intravenous (IV) 8 mg dexamethasone, while Group B patients were administered with subhypnotic dose of propofol (0.5 mg/kg, IV).

**Figure 1:**
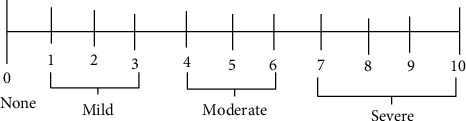
Numeric rating scale (NRS).

Thereafter, in the postanesthesia care unit (PACU), patients were asked to report the severity and occurrence of nausea or vomiting, as well as their need for additional antiemetics based on the 11-point NRS score, once fully able to respond to verbal commands. Patients were fully aware to classify PONV severity as no for score 0, mild for 1–3, moderate for 4–6, and severe for 7–10 with propensity to vomiting (Figure 1). Then trained professionals had assessed and recorded the severity score (Figure 1). Rescue antiemetic was given at recommended dose intravenously to the patients during active vomiting or with NRS score of 4 and above. The incidence and severity of PONV and associated adverse effects were documented at the 6th hour, 12th hour, and 24th hour after the administration of dexamethasone and propofol. In addition, the requirement of rescue antiemetics in the overall 24 hours was documented.

Statistical analysis was done using SPSS version 20 software. Data distributions were tested by using Shapiro–Wilk test while homogeneity of variance was assessed with Levene's test for equality of variance. Unpaired Student's *t*-test and Mann–Whitney test were used for comparison of numerical variables between study groups. Frequency and percentage were used to describe categorical variables, and the statistical difference between groups was tested using the chi-square test. A *p* value < 0.05 with 95% confidence interval and a power of 80% were considered statistically significant.

## 3. Results

### 3.1. Sociodemographics and Preoperative Characteristics

The majority of patients were ASA I (82.5%) and females (51.25%). There was no statistically significant difference between the dexamethasone and propofol groups in terms of age, oral intake times, sex, body mass index (BMI), type of surgery, ASA status, or duration of anesthesia and surgery (Table 1).

### 3.2. Intraoperative Characteristics

Thiopentone (55%) and both tramadol and diclofenac (48.75%) were the most commonly used induction analgesic agents, respectively, with no statistically significant difference in the intraoperative variables between the two groups (Table 2).

### 3.3. Incidence of PONV and Rescue Antiemetic Use over the Follow-Up Period

**Figure 2:**
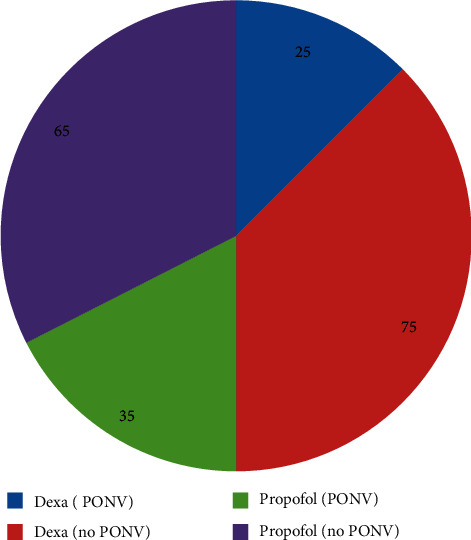
Incidence of PONV (%) from 0 to 24 hours. Dexa: dexamethasone; PONV: postoperative nausea and vomiting.

In the dexamethasone group, the requirement of rescue antiemetic treatment (0% versus 5%, *p*=0.02) and incidence of PONV (0% versus 22.5%, *p* ≤ 0.001) were statistically significantly lower compared to patients enrolled in propofol group over the 12th–24th hours (Table 3). As indicated in Figure 2, the overall incidence of PONV was higher in patients administered with propofol than those administered with dexamethasone. In propofol group, 35% of the cases experience PONV, while the incidence of PONV was 25% in dexamethasone group.

### 3.4. Severity of Nausea

**Figure 3:**
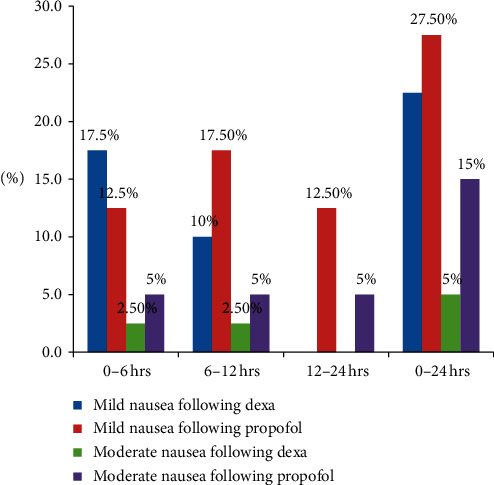
Comparison of postoperative nausea severity using 11-point NRS score (0–10).

The incidence of nausea in dexamethasone group gradually decreased in the subsequent time intervals over the 24-hour follow-up period. Of all participants, 11 (27.5%) of those receiving propofol reported mild nausea as opposed to 9 (22.5%) of those receiving dexamethasone, while 6 (15%) of propofol recipients experienced moderate nausea compared with 2 (5%) of their dexamethasone receiving counterparts. None of the participants experienced severe nausea in the overall 24-hour follow-up period. These findings were statistically significant in the 12–24 hr postoperative period (*p*=0.012) (Figure 3).

### 3.5. Reported Side Effects

**Figure 4:**
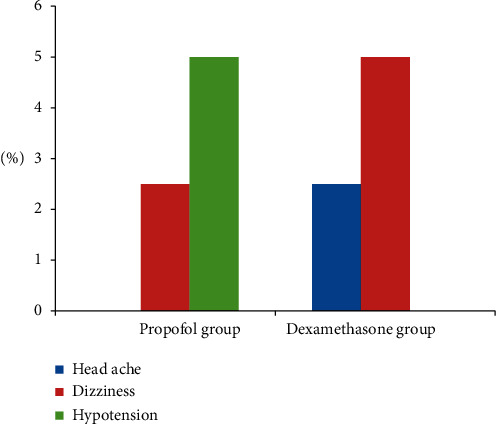
Associated side effects observed within 24 hours after taking antiemetics.

During the overall follow-up, 2.5% of patients in propofol group and 5% patients in dexamethasone group complained of dizziness, whilst 3% of patients in propofol group reported sedation, but there was no hypoxia and difficulty of breathing and 2.5% of participants in the dexamethasone group experienced headache (Figure 4).

## 4. Discussion

ENT surgery has been associated with high incidence of PONV, especially in patients without prophylactic antiemetic agents [2, 4, 23]. During a surgical procedure, serotonin is released from the gastrointestinal tract from enterochromaffin cells and binds to visceral receptors of the 5-HT 3 subtype, causing stimulation of vagal afferents in the gastrointestinal tract to conduct impulses that reach the Chemoreceptor Trigger Zone (CTZ) located on the dorsal surface of the medulla oblongata at the caudal end of the fourth ventricle. CTZ stimulation due to the arrived stimulus will lead to PONV [24].

In our study, the overall incidence of PONV was higher in the propofol group than dexamethasone group (35% versus 25%) with statistical significance in the 12th to 24th hour period (*p* < 0.002). The requirement for rescue antiemetics was relatively lower in dexamethasone group.

Glucocorticoids have been widely used to prevent PONV during chemotherapy use or general anesthesia. Although the antiemetic mechanism is not clearly understood, scientific evidence suggests that dexamethasone reduces production and release of 5-HT and decreases permeability across the Blood-Brain Barrier (BBB) thereby lowering the amount of 5-HT available to chemical sensors [18, 25]. However, the use of dexamethasone may be associated with increased risk of infection, reduced wound healing, and interference with the functioning of adrenal glands through negative feedback-mediated reduction of endogenous steroid synthesis.

In dexamethasone group, the incidence of PONV was low in the periods of 0–6 hrs and 6–12 hrs, while there are no participants encountering nausea and vomiting in the latter period (i.e., 12–24 hrs). Indeed, low or no PONV with dexamethasone could be attributable to its fast onset and long duration of action (i.e., 72 hours) [26].

Similarly, a bolus dose of propofol produced preventive antiemetic effects in patients undergoing ENT surgery in this study. However, the protective action of propofol was decreased in the subsequent time intervals as compared to dexamethasone group. In line with this result, the requirement of rescue antiemetic therapy was lower in the dexamethasone than propofol recipients (5% versus 12.5%, *p* < 0.23) over the 24 hr period. Statistically significant results were obtained at the 12th to 24th hour time period (*p* < 0.044). Studies suggest that effective concentration of propofol is better maintained in IV infusion rather than bolus dosing to prevent PONV [27, 28]. The antiemetic effect of propofol is attributed to modulation of subcortical pathways to inhibit nausea or its direct depressant action on the vomiting center [29]. The results of our study are consistent with other studies, conducted in different settings, in terms of antiemetic rescue therapy requirements and trends of dexamethasone preventive effect [22, 23, 25, 30, 31].

## 5. Conclusion

In summary, dexamethasone produced better PONV protection than propofol in all time intervals. Nevertheless, there are some limitations in this study. For instance, values were not statistically significant in most of the time intervals, which may reflect the small sample size in both groups. Moreover, placebos were not used to check the outcomes of drug treatment and without treatment. Therefore, we recommend future research with a larger sample size and studies with placebo group. Further, we recommend a randomized controlled trial be conducted to further validate these findings.

## Data Availability

The data for this study were collected from Tikur Anbessa Specialized Hospital and Yekatit 12th Hospital. The collected data are available in SPSS and data collection tools.

## Abbreviations

ASA: American Society of Anesthesiology
BMI: Body mass index
ENT: Ear, nose, and throat
5HT3: 5-Hydroxytryptamine receptors
ICU: Intensive care unit
NRS: Numeric rating score
PONV: Postoperative nausea and vomiting.

## Supplementary Materials

Supplementary Materials Table 1: sociodemographics and preoperative characteristics of study participants. Table 2: intraoperative characteristics of participants. Table 3: incidence of PONV and requirement of rescue antiemetic.
