# Supplementary material for: Effectiveness of Propofol versus Dexamethasone for Prevention of Postoperative Nausea and Vomiting in Ear, Nose, and Throat Surgery in Tikur Anbessa Specialized Hospital and Yekatit 12th Hospital, Addis Ababa, Ethiopia
Source: Anesthesiol Res Pract. 2020 Sep 7;2020:4258137. doi: 10.1155/2020/4258137 (PMC7492878; doi:10.1155/2020/4258137)
Supplement: Supplementary Materials — Table 1: sociodemographics and preoperative characteristics of study participants. Table 2: intraoperative characteristics of participants. Table 3: incidence of PONV and requirement of rescue antiemetic. [file 4258137.f1.docx]

**Table 1:** Sociodemographics and preoperative characteristics of study participants

| Variables | Propofol group N=40 | Dexamethasone group N=40 | P value |
| --- | --- | --- | --- |
| Age (mean ±SD) | 33.57±10.9 | 32.9±10.6 | 0.59 |
| Sex (Female /male n%) | 55%**/**45% | 47.5%/52.5% | 0.66 |
| ASA(I/ II n%) | 80% /20% | 87.5% /12.5% | 0.54 |
| BMI (median and IQR) | 20.5(19-22.4) | 20.7(19.5-21.6) | 0.89 |
| NPO times(hrs) (median and IQR) | 10(9-10.5) | 10(9-10) | 0.82 |
| Type of surgery  Middle ear surgery | 18(45%) | 17(42.5%) | 0.34 |
| Nasal surgery | 9(22.5%) | 8(20%) | 0.49 |
| Throat surgery | 13(32.5%) | 15(37.5%) | 0.24 |
| Total | 40 | 40 |  |

**Abbreviations:** n(%), number and percentage; NPO, nothing by mouth; ASA, [American Society of](https://www.asahq.org/) [Anesthesiologists](https://www.asahq.org/); BMI, Body Mass index; IQR, Interquartile Range

**Table 2:** Intraoperative characteristics of participants

| Variables | Propofol group N=40 | Dexamethasone group N=40 | P value |
| --- | --- | --- | --- |
| Duration of surgery(min) (Median and IQR) | 65(45-110) | 57(50-80) | 0.35 |
| Duration of anesthesia(min) Median and  IQR | 80(57.5-120) | 65(60-85) | 0.27 |
| Estimated intra operative blood loss (ml)  Median and IQR | 150(100-300) | 150(100-250) | 0.99 |
| Total fluid replaced(ml) Median and IQR | 800(500-1000) | 700(525-1000) | 0.29 |
| Induction agents  Ketamine | 20(50%) | 16(40%) |  |
|  |  |  | 0.50 |
| Thiopentone | 20(50%) | 24(60%) | 0.35 |

| Intraoperative analgesia  Fentanyl | 5(12.5%) | 6(15%) | 0.64 |
| --- | --- | --- | --- |
| Morphine | 10(25%) | 6(15%) | 0.27 |
| Pethidine | 6(15%) | 8(20%) | 0.66 |
| Tramadol and diclofenac | 19(47.5%) | 20(50%) | 0.71 |

Abbreviations: Inter Quartile Range(IQR); Milliliter(ml); Number (percentage), **n**(%)

**Table 3:** Incidence of PONV and requirement of rescue anti-emetic

| Scale of PONV | Propofol group  (N=40) | Dexamethasone group  (N=40) | X2 | P value |
| --- | --- | --- | --- | --- |
| Nausea (n, %) 0-6 hours | 5(12.5%) | 6(15%) | .105 | 0.74 |
| 6-12 hours | 6(15%) | 4(10%) | .457 | 0.49 |
| 12-24 hours | 6(15%) | 0% | 6.486 | 0.026* |
| Vomiting (n, %) 0-6 hours | 2(5%) | 1(2.5%) | .346 | 0.55 |
| 6-12 hours | 3(7.5%) | 2(5%) | 0.00 | 1.000 |
| 12-24 hours | 3(7.5%) |  | 0% | 4.276 |
| Rescue antiemetic (n, %) 0-  6 hours | 1(2.5%) | 1(2.5%) |  | 1.00 |
| 6-12 hours | 3(7.5%) | 0% | .384 | 0.241 |
| 12-24 hours | 2(5%) | 0(0%) | 5.356 | 0.021* |
| Total PONV (n, %) 0-6  Hours | 7(17.5%) | 7(17.5%) |  | 1.00 |
| 6-12 hours | 9(22.5%) | 6(15%) | .738 | 0.39 |
| 12-24 hours | 9(22.5%) | 0 | 8.01 | 0.002 |
| **0–24 hours**  Total PONV (𝑛, %) | 14(35%) | 10(25%) |  |  |
